# Senescent Cells: A Potential Target for New Cancer Therapies in Older Oncologic Patients

**DOI:** 10.3390/cancers13020278

**Published:** 2021-01-13

**Authors:** Baukje Brattinga, Barbara L. van Leeuwen

**Affiliations:** Department of Surgery, University Medical Center Groningen, University of Groningen, Hanzeplein 1, 9700 RB Groningen, The Netherlands; b.brattinga@umcg.nl

Cellular senescence is a complex process and is one of the key elements of ageing. In senescent cells, there is an irreversible growth arrest in cell proliferation that is induced by a range of stimuli, like DNA damage, telomere shortening, cellular stress, and oncogene activation. Senescent cells are characterized by changes in gene expression, in particular the activation of the senescence-associated secretory phenotype (SASP), which can result in different effects [1,2]. Short term, the senescence response induces an inflammatory response and promotes tissue repair and suppresses tumor genesis. Long term, the release of SASP can have a pro-tumorigenic role and can induce tumor genesis, chronic inflammation, and tissue ageing. In this state, senescent cells have not reached the state of persistent cell-cycle arrest [1,2]. As cellular senescence is related to tissue ageing, it can cause physical dysfunction and frailty.

Many widely used cancer therapies, like cytostatic drugs, immunotherapy, and radiotherapy, induce cellular senescence. This process is also called therapy-induced senescence. When the damage of the therapy is severe, this will lead to cell death. If the damage through chemo-, immune-, or radiotherapy is not lethal, cells will enter a chronic state of cellular senescence. There is evidence that tumor cells can re-enter the cell cycle after therapy-induced senescence, and this process can have detrimental effects. The release of SASP from senescent cancer cells may eventually lead to tumor recurrence or progression [3].

How changes in senescent tumor cells eventually cause tumor recurrence or progression is largely unknown. Different pathways are described. Perrigue et al. studied the process of senescent tumor cells reprogramming into cancer stem cells. The effects of treating fibroblast and cancer cells with a stem cell medium were observed and the process of oxidative stress and reprogramming into senescent cancer cells was studied. Factors associated with the cancer stem cell phenotype were also identified [4]. These factors associated with the remodeling of senescent cells into cancer stem cells could be predictive of poor outcome after cancer treatment and possible targets for new therapies. Liebig et al. found that the overexpression of the RNA-binding protein HuR in melanoma cell lines is an important factor in the pathway regulation of BRAF-induced senescence and thereby in the development and tumor progression of melanoma [5]. These studies give new insights into cellular changes in senescent tumor cells that may lead to the detrimental effects of tumor progression and tumor recurrence.

The potential of a new group of drugs called senotherapies is discussed in different review articles. Senolytic and senostatic drugs can interact with senescent cells by selectively destroying senescent cells or by selectively suppressing the release of SASP and in doing so inhibit the function of senescent cells, with the goal of eliminating cancer cells. Senolytic drugs might improve physical function and survival at old age as well [6]. Wyld et al. describe navitoclax, fisetin, dasatinib/quercetin, Hsp90 inhibitors, and metformin as potential senolytic and senostatic drugs [7]. Also, panobinostat, authophagy modulators, and cardiac glycosides are discussed by Saleh et al. as potential senotherapies [3]. Milczarek describes the potential of implementing treatments indicting senescence for the specific group of patients with breast cancer [8]. Nitric oxide (NO) is described by Mabrouk et al. as a potential cancer therapy. NO modulates the expression of SASP, induces apoptosis, and activates the immune system of senescent cells. Pharmacologically active substances that release NO, also called NO donors, are described in different trials as promising senotherapies in combination with chemo- or radiotherapy to treat cancer [9]. These new potential drugs could be innovative therapeutic approaches for the specific category of patients with tumor recurrence or tumor growth after chemo-, immune-, or radiotherapy.

Since the worldwide population is ageing, the incidence of older cancer patients is rapidly increasing [10]. With aging comes a decline in function and the development of degenerative diseases like cancer. Older frail patients are especially at greater risk for complications and poor outcome after cancer treatment, and are a distinct group when it comes to challenges for optimal treatment choice [11]. There is increasing evidence that cellular senescence could play a role as a prognostic marker for frailty and cancer, and that cellular senescence could be a target for new cancer therapies as senolytic and senostatic drugs.

Further research is needed to evaluate the effect of these drugs. If senotherapies can reduce frailty and can reduce the risk of tumor recurrence and progression in therapy-induced senescent cells, this would be of great value for clinical practice of cancer treatment in older frail patients. Important challenges for future research to address include finding drug agents that selectively eliminate or suppress senescent tumor cells without severe side effects for frail older patients.

In summary, this Special Edition of *Cancers* represents a collaborative and international effort that describes current insights into cellular senescence and innovative possibilities for curative cancer treatment. This collection of articles may help in reaching the ultimate goal of improving outcomes after cancer treatment for (older) oncologic patients, and in reducing the likelihood of cancer relapse after treatment.

## Data Availability

Not applicable.

## References

[1] Van Deursen J.M. (2014). The role of senescent cells in ageing. Nature.

[2] Campisi J. (2013). Aging, Cellular Senescence, and Cancer. Annu. Rev. Physiol..

[3] Saleh T., Bloukh S., Carpenter V.J., Alwohoush E., Bakeer J., Darwish S., Azab B., Gewirtz D.A. (2020). Therapy-Induced Senescence: An “Old” Friend Becomes the Enemy. Cancers.

[4] Perrigue P.M., Rakoczy M., Pawlicka K.P., Belter A., Giel-Pietraszuk M., Naskręt-Barciszewska M., Barciszewski J., Figlerowicz M. (2020). Cancer Stem Cell-Inducing Media Activates Senescence Reprogramming in Fibroblasts. Cancers.

[5] Liebig J.K., Kuphal S., Bosserhoff A.K. (2020). HuRdling Senescence: HuR Breaks BRAF-Induced Senescence in Melanocytes and Supports Melanoma Growth. Cancers.

[6] Xu M., Pirtskhalava T., Farr J.N., Weigand B.M., Palmer A.K., Weivoda M.M., Inman C.L., Ogrodnik M.B., Hachfeld C.M., Fraser D.G. (2018). Senolytics improve physical function and increase lifespan in old age. Nat. Med..

[7] Wyld L., Bellantuono I., Tchkonia T., Morgan J., Turner O., Foss F., Jayan G., Danson S., Kirkland J.L. (2020). Senescence and Cancer: A Review of Clinical Implications of Senescence and Senotherapies. Cancers.

[8] Milczarek M. (2020). The Premature Senescence in Breast Cancer Treatment Strategy. Cancers.

[9] Mabrouk N., Ghione S., Laurens V., Plenchette S., Bettaieb A., Paul C. (2020). Senescence and Cancer: Role of Nitric Oxide (NO) in SASP. Cancers.

[10] Pilleron S., Sarfati D., Janssen-Heijnen M., Vignat J., Ferlay J., Bray F., Soerjomataram I. (2019). Global cancer incidence in older adults, 2012 and 2035: A population-based study. Int. J. Cancer.

[11] Partridge J.S.L., Harari D., Dhesi J.K. (2012). Frailty in the older surgical patient: A review. Age Ageing.

